# Mycotoxin Contamination of Feeds and Raw Materials in China in Year 2021

**DOI:** 10.3389/fvets.2022.929904

**Published:** 2022-06-30

**Authors:** Wei Hao, Anping Li, Jinyong Wang, Gang An, Shu Guan

**Affiliations:** ^1^Department of Animal Nutrition and Health, DSM (China) Co., Ltd, Shanghai, China; ^2^Department of Animal Nutrition and Health, DSM Singapore Industrial Pte Ltd, Singapore, Singapore

**Keywords:** mycotoxin contamination, maize, feed, raw material, China

## Abstract

In this research, we performed a large-scale survey of mycotoxin contamination in several feed commodities and assessed regional differences in mycotoxin occurrence in maize across China in 2021. Concentrations of aflatoxins, zearalenone (ZEN), fumonisins, and trichothecenes type B were analyzed in 2,643 raw material and compound feed samples collected from eight provinces. Generally, trichothecenes type B, fumonisins, and ZEN were most prevalent and detected in averages of positive concentrations at 1,167, 1,623, and 204 μg/kg, respectively. In the new season maize, samples were also seriously infested with trichothecenes type B, fumonisins, and ZEN, and their averages of positive concentrations were 1,302, 2,518, and 225 μg/kg, respectively. Wheat was commonly contaminated with trichothecenes type B and ZEN, and the highest concentration levels of trichothecenes type B, fumonisins, and ZEN were all detected in the samples from maize by-products. Among the different geographical regions, distinct trends were observed in new season maize. Samples from Shandong province were highly contaminated with trichothecenes type B, fumonisins, and ZEN, while special attention should be paid to aflatoxins and fumonisins in Anhui and Jiangsu provinces in East China. In addition, the present survey showed that compound feeds and raw materials are commonly contaminated by multiple mycotoxins. Trichothecenes type B and ZEN concentrations were correlated significantly in this survey.

## Introduction

In recent years, driven by increased demands for livestock production, in addition to traditional crops, their by-products, such as bran, dried distillers' grains and soluble fraction (DDGS), and soybean meal, are widely utilized in the manufacture of animal feeds nowadays in China. Several factors play essential roles in mycotoxins contamination, such as extreme weather conditions around harvesting, poor hygiene practices during transportation, storage, and manufacture of the by-products. This has led to increased awareness of mycotoxin issues.

Mycotoxins can be easily found in fungi-infested feed raw materials, and the most common toxin-synthesizing fungi are *Aspergillus, Fusarium, Penicillium*, and *Alternaria* spp. (1). There are statistically more than 300 known mycotoxins described (2), and among these, aflatoxins, zearalenone (ZEN), trichothecenes, and fumonisins are the most common toxins around the world (3). China, one of the countries with serious mycotoxin contamination, has placed stricter regulations on mycotoxins in feeds and raw materials in recent years.

Aflatoxins are mainly produced by strains of *Aspergillus flavus* and *Aspergillus parasiticus*, and are suggested to have carcinogenic and mutagenic effects on animals (4). Among the feed materials, wheat, maize, and rice are mostly affected by aflatoxins (5). To control the negative effects, China has regulated aflatoxin B1 (AFB1) with ranges from 10 to 50 μg/kg for raw materials, and 10 to 30 μg/kg for finished feeds.

Zearalenone, largely produced by *Fusarium graminearum* and *Fusarium Culmorum*, is an estrogenic mycotoxin, causing reproductive disorders and estrogenic disruption in humans and animals (6). The maximum levels of ZEN in China are regulated to range from 500 to 1,500 μg/kg for feed materials and are limited to 500, 150, and 100 μg/kg for ruminant concentrate supplement, piglet feed, and young sow feed, respectively.

Trichothecenes comprise a large class of fungal metabolites and are subdivided into four types. Trichothecenes type B, which is normally represented by Deoxynivalenol (DON), is largely produced by *F. graminearum, F. culmorum*, and *Fusarium nivale*. Trichothecenes type B are reported to occur worldwide and can cause negative symptoms involving vomiting, feed refusal, skin damage, and hemorrhage (7, 8), especially in swine. The maximum concentrations of DON are regulated to be 5,000 μg/kg in feed materials, and 3,000 μg/kg in complementary and compound feeds in China.

*Fusarium moniliforme, Fusarium verticillioides*, and *Fusarium proliferatum* are the main producers of fungi for fumonisins (6). The toxins have been reported to cause sphingolipid biosynthesis disruption and leukoaraiosis (9), and Fumonisin B1 (FB1) is recognized to be the most strong and predominant one. The maximum levels for the sum of FB1 and FB2 are 60,000 μg/kg for feed materials and range from 5,000 to 50,000 μg/kg for complementary and compound feeds, depending on animal species.

Multiple regional survey programs of mycotoxin contamination in materials and animal finished feeds have been conducted all over the world (10–14). Due to environmental conditions, such as temperature and precipitation, which are key determinants of the contamination situation (15), mycotoxin contamination of different regions shows specific pollution patterns. However, detailed mycotoxin prevalence data from feed material and compound feed in China have been lacking. In this study, we analyzed the occurrence of aflatoxins, ZEN, trichothecenes, and fumonisins in 2,643 samples collected from different regions of China in 2021. We compared mycotoxin occurrences in new season maize from different regions and analyzed the variation of mycotoxin concentrations in different feed commodities. Through the collection of data, we can provide vital information on the mycotoxin contamination of feeds, and further support the risk management of the mycotoxins relevant to animal health in China.

## Materials and Methods

### Samples and Mycotoxin Standards

A total of 2,643 samples of feed raw materials and finished feeds were collected from 8 provinces of China during the period from January 2021 to December 2021. The dataset comprised 966 samples of finished feed (350 in swine feed, 434 in poultry feed, 148 in Total Mixed Ration (TMR), and 34 in concentrate supplement), 910 samples of maize, 380 samples of other raw materials (226 in wheat, 65 in bran, 65 in soybean meal, 13 in cottonseed gluten meal, 12 in rice bran meal, and eight in peanut meal), 58 samples of maize by-product (27 in DDGS, 20 in corn gluten meal, and 11 in sprayed corn husk), and 329 samples of grasses (243 in silage, 64 in oat grass, 16 in alfalfa, and six in soybean hull). Complete notes with details surrounding the circumstances of the samples, including temperature, moisture, and water content, were submitted with the samples. Then, 1 kg of original samples was collected and kept at 4°C before being transported to the analytical Romer Labs (Wuxi, China). Sampling, milling, and homogenization of a 100 g representative sub-sample were performed as described by Kovalskyet al. (10).

### Mycotoxin Analysis

In total, 1,649 samples were analyzed using liquid chromatography-tandem mass spectrometry (LC-MS/MS), a multi-mycotoxin analysis method. This method was used in particular for more complex matrices, such as DDGS, finished feed, silage, and TMR. By applying this method, 18 mycotoxins, including four kinds of aflatoxins (AFB1, AFB2, AFG1, and AFG2), ZEN, five kinds of trichothecenes type B (DON, 3-Acetyl-Deoxynivalenol, 15-Acetyl-Deoxynivalenol, Nivalenol, and Fusarenon X), four kinds of trichothecenes type A (H-2 toxin, HT-2 toxin, Diacetoxyscirpenol, and Neosolaniol), three kinds of fumonisins (FB1, FB2, and FB3), and OTA, can be detected simultaneously. For the purpose of data analysis, non-detect levels are based on the limits of detection (LOD) of the test method for each toxin. LODs were 0.5 μg/kg for AFB1, AFB2, AFG1, and AFG2, 10 μg/kg for ZEN, 10 μg/kg for DON, 10 μg/kg for FB1, FB2, and FB3, 0.5 μg/kg for OTA, and 10 μg/kg for T-2 toxin, respectively.

Procedures of sample preparation and instrumental parameters were performed according to the methods of Malachová et al. (16) and 11, with slight modifications which are listed as follows. Samples preparation: a total of 25 g of the ground sample was extracted with 100 ml of acetonitrile/water (50:50, v/v) in a blender for 1 h, and after filtration, 2 ml of the extraction solvent was added with 100 μl of acetic acid. Then, 750 μl of the mixture was subsequently applied to a Mycospin^TM^ 400 column (Romer Labs. Inc., Austria) for purification. The sample solution was then centrifuged for 1 min at 10,000 rpm and 20 μl of the supernatant was injected into the LC–MS/MS system without further pre-treatment. Instrument parameters: detection and quantification were performed with a QTrap 5500 MS/MS system (Applied Biosystems, Foster City, CA, US) equipped with a TurboV electrospray ionization (ESI) source and a 1,290 series UHPLC system (Agilent Technologies, Waldbronn, Germany). Chromatographic separation was performed at 40°C on a Gemini^®^ C18-column, 150 mm × 4.6 mm i.d., 5 m particle size, equipped with a C18 security guard cartridge, 4 mm × 3 mm i.d. (Phenomenex, Torrance, CA, US). The flow rate was 1 ml/min. Elution was carried out in the binary gradient mode. Both mobile phases contained 2 mM ammonium acetate and eluent A and eluent B were composed of water/acetic acid 199:1 (v/v) and methanol/acetic acid 199:1 (v/v), respectively. After an initial time of 1 min at 90% A, the proportion of B was increased linearly to 97% within 14 min and held for 1 min, and then the proportion of A was increased back to 90% for the next 5 min for column re-equilibration. Electrospray ionization (ESI) was performed in the scheduled selected reaction monitoring (sSRM) mode both in positive and negative polarities in two separate chromatographic runs. The settings of the ESI source were as follows: positive polarities source temperature 650°C, negative polarities source temperature 600°C, curtain gas 35 psi, collision gas medium, ion-spray voltage −4,500 and +5,000 V, respectively, ion source gas 1 60 psi, and ion source gas 2 65 psi.

The remaining 994 samples of feed raw materials were detected using the method of enzyme linked immunosorbent assay (ELISA). The LODs were 2 μg/kg for AFB1, 25 μg/kg for ZEN, 250 μg/kg for DON, and 250 μg/kg for fumonisins, respectively. Procedures of sample preparation and analyses were performed with commercially available test kits (AgraQuant^®^Assay, Romer Labs Diagnostic GmbH, Austria) according to their operating instructions.

For all analyzed samples, when the mycotoxin levels were higher than the following values, the samples can be defined as mycotoxin positive: the concentration threshold was 1 μg/kg for the sum of AFB1, AFB2, AFG1, and AFG2, 32 μg/kg for ZEN; 50 μg/kg for trichothecenes type B, and 100 μg/kg for fumonisins. Correlations between mycotoxin contaminations were analyzed with the ggpairs in the ggally package (17) using R software, version 3.3.0 (18). Results below the LODs were treated as zero values in the correlation analysis.

## Results

### General Mycotoxin Occurrence

**Figure 1:**
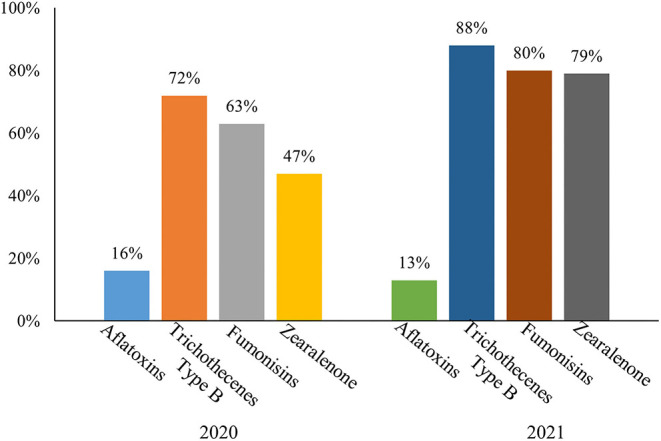
Positive rates of main mycotoxins generally in China in 2021 and 2020.

In total, 2,643 samples collected throughout China were analyzed for aflatoxins, ZEN, trichothecenes type B, and fumonisins. The *Fusarium* mycotoxins trichothecenes type B, fumonisins, and ZEN were most prevalent and detected in 88, 80, and 79% of all samples (Figure 1), with an average of positive concentrations at 1,167, 1,623, and 204 μg/kg, respectively. Aflatoxins were detected in 13% of all samples. The average of positive concentrations, max concentrations, and positive rates of trichothecenes type B and fumonisins were higher, whereas aflatoxins showed relatively lower contamination levels in 2021 compared with 2020. The average of positive and max concentrations of ZEN showed little variation between the years, while its positive rate was significantly increased (Table 1).

**Table 1:** The occurrence of main mycotoxins generally in China in the year 2021 and year 2020.

**Item**		**Aflatoxins**	**Trichothecenes type B**	**Fumonisins**	**Zearalenone (ZEN)**
2021 (2643)^a^	Average of positives (μg/kg)	17	1,167	1,623	204
	Maximum (μg/kg)	331	20,213	41,700	10,467
2020 (1610)^a^	Average of positives (μg/kg)	34	651	1,564	206
	Maximum (μg/kg)	482	10,426	30,872	11,245

a *Samples number*.

### Occurrence of Mycotoxins in New Season Maize

**Figure 2:**
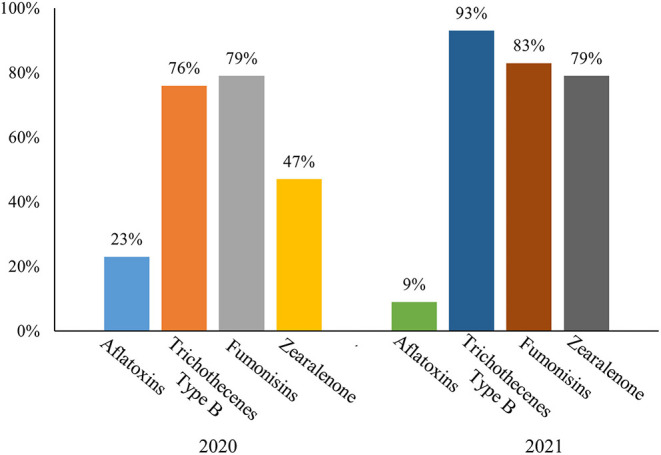
Positive rates of main mycotoxins of new season maize in China in 2021 and 2020.

In 2021, 347 samples of new season maize were collected right after harvesting in autumn, mainly from medium- and large-scale feed mills and livestock farms. In general, mycotoxins of ZEN, trichothecenes type B, and fumonisins were the most dominating. Their contamination levels were increased, with an average of positive concentrations of 225, 1,302, and 2,518 μg/kg, and positive rates of 79, 93, and 83%, respectively (Figure 2). Aflatoxins showed the opposite trend, and their positive rate was only 9% (Table 2).

**Table 2:** The occurrence of main mycotoxins of new-season maize in China in the year 2021 and year 2020.

**Item**		**Aflatoxins**	**Trichothecenes Type B**	**Fumonisins**	**Zearalenone (ZEN)**
2021 (910)^a^	Average of positives (μg/kg)	35	1,302	2,518	225
	Maximum (μg/kg)	331	12,808	38,563	2,883
2020 (214)^a^	Average of positives (μg/kg)	52	735	3,811	112
	Maximum (μg/kg)	482	4,670	23,480	1,572

a *Samples number*.

To elucidate the regional trends of mycotoxin occurrence of new season maize, the whole dataset was broken down into seven province sub-datasets in four geographic regions of China, with Jiangsu and Anhui provinces combined into one. For each of these sub-datasets, the prevalence and average of positive concentrations of aflatoxins, fumonisins, ZEN, and trichothecenes type B are shown in Table 3.

**Table 3:** Mycotoxin occurrence of new season maize of different regions in China in 2021.

**Mycotoxins**	**Sample number**	**Positive samples**		**Average of positives** **(μg/kg)**	**Maximum (μg/kg)**
		**Number**	**%**		
**Heilongjiang province, Northeast China**					
Aflatoxins	27	3	11	4	8
Trichothecenes Type B	27	22	81	1,251	3,172
Fumonisins	27	16	59	1,807	5,021
Zearalenone (ZEN)	27	16	59	195	726
**Jilin province, Northeast China**					
Aflatoxins	28	2	7	13	19
Trichothecenes Type B	28	27	96	1,067	2,322
Fumonisins	28	27	96	1,460	4,180
ZEN	28	26	93	68	181
**Liaoning province, Northeast China**					
Aflatoxins	40	0	0	0	0
Trichothecenes type B	40	40	100	1,524	3,528
Fumonisins	40	39	98	2,097	8,360
ZEN	40	39	98	141	598
**Hebei province, North China**					
Aflatoxins	72	9	13	17	31
Trichothecenes type B	72	61	85	1,334	3,439
Fumonisins	72	64	89	3,154	18,320
ZEN	72	55	76	226	939
**Shandong province, East China**					
Aflatoxins	81	5	6	19	47
Trichothecenes type B	81	70	86	**1,954**	**6,849**
Fumonisins	81	74	91	4,665	17,840
ZEN	81	71	88	**722**	**3,017**
**Henan province, Central China**					
Aflatoxins	56	35	63	47	211
Trichothecenes type B	56	53	95	768	2,430
Fumonisins	56	55	98	3,541	14,505
ZEN	56	48	86	144	770
**Anhui and Jiangsu provinces, East China**					
Aflatoxins	43	39	91	77	331
Trichothecenes type B	43	34	79	548	2,310
Fumonisins	43	42	98	4,430	11,776
ZEN	43	22	51	105	442

For Northeast China, the positive rates of aflatoxins in Heilongjiang, Jilin, and Liaoning provinces were relatively at low levels. On the other hand, trichothecenes type B were frequently detected and showed average positive values above 1,000 μg/kg. In Liaoning province, trichothecenes type B showed 100% prevalence, and the positive concentration almost doubled than that of last year (data not shown) with an average of 1,524 μg/kg. Fumonisins were detected with positive rates of 59, 96, and 98%, respectively, in Heilongjiang, Jilin, and Liaoning provinces, and the averages of positives were slightly lower than or equivalent to 2,000 μg/kg. The positive rates of ZEN have increased since the year 2020, and the average in Heilongjiang has increased to 195 μg/kg. About 11 and 5% of samples from Heilongjiang and Liaoning provinces, respectively, exceeded the regulated ZEN value of maize in China.

In the Hebei province of North China, aflatoxins were detected in 13% of samples at an average positive concentration of 17 μg/kg. Approximately, 80% of the samples were found to be contaminated with trichothecenes type B, fumonisins, and ZEN, and their averages were 1,334, 3,154, and 226 μg/kg, respectively. Approximately, 14% of samples exceeded the regulated value of ZEN in China.

As for East China Shandong province, aflatoxins were detected in only 6% of the samples. Trichothecenes type B and ZEN were the most dominant of all regions analyzed and detected in 86 and 88% of the samples, with averages of positive concentrations of 1,954 and 722 μg/kg, respectively. Consequently, 41 and 15% of these samples exceeded the maximum limits for ZEN and trichothecenes type B, respectively, in the maize of China.

For the Henan province of Central China, aflatoxins showed a positive rate as high as 63%, with an average of 47 μg/kg, which was the highest average of positive concentrations among all regions. In total, 21% of samples exceeded the regulated value for AFB1. Trichothecenes type B and fumonisins were detected in more than 95% of the samples, and ZEN was detected in 86% of the samples.

For Jiangsu and Anhui provinces in East China, 91% of the samples showed positive aflatoxins contamination, and 42% exceeded the regulated value. The positive rate of ZEN in Jiangsu and Anhui was the lowest of all regions. Trichothecenes type B showed a positive rate of 79%, with a relatively low average positive concentration of 548 μg/kg.

### Occurrence of Mycotoxins in Different Feed Raw Materials

Different feed raw materials presented different patterns of mycotoxin contamination (Table 4). In 910 samples of maize, trichothecenes type B, fumonisins, and ZEN were detected in a high fraction at positive rates of 93, 83, and 79%, respectively, and the positive averages were 1,302, 2,518, and 225 μg/kg, respectively. Only 3% of the samples were detected with aflatoxins in maize, however, their average reached a relatively high level of 35 μg/kg. In wheat, ZEN was detected in 49% and trichothecenes type B was detected in 68% of the samples with an average of positive concentrations of 1,103 μg/kg. In soybean meal, ZEN was prevalent. It was detected in a high fraction (70%) at a low average of positives of 47 μg/kg. Peanut meal has a high risk of aflatoxins contamination. Aflatoxins were found positive in 100% of samples of peanut meal and the average was 191 μg/kg, with a relatively high-risk level. Trichothecenes type B were detected positive in 94% of samples of bran at an average positive concentration of 1,190 μg/kg. Rice bran meal was mainly contaminated with trichothecenes type B and ZEN. Cottonseed gluten meal was mainly contaminated with fumonisins at a positive rate of 68%.

**Table 4:** Mycotoxin occurrence of different feed raw materials in China in 2021.

**Mycotoxin**	**Sample number**	**Positive samples**		**Average of positives (μg/kg)**	**Maximum (μg/kg)**
		**Number**	**%**		
**Maize**					
Aflatoxins	910	27	3	35	331
Trichothecenes Type B	910	846	93	1,302	12,808
Fumonisins	910	755	83	2,518	38,563
Zearalenone (ZEN)	910	719	79	225	2,883
**Wheat**					
Aflatoxins	226	2	1	2	3
Trichothecenes type B	226	154	68	1,103	7,425
Fumonisins	226	61	27	347	910
ZEN	226	111	49	79	619
**Soybean meal**					
Aflatoxins	56	3	5	2	3
Trichothecenes type B	56	5	9	46	113
Fumonisins	56	6	11	19	39
ZEN	56	39	70	47	88
**Peanut meal**					
Aflatoxins	8	8	100	191	440
Trichothecenes type B	8	1	13	18	18
Fumonisins	8	4	50	33	69
ZEN	8	1	13	14	14
**Bran**					
Aflatoxins	65	3	5	3	4
Trichothecenes type B	65	61	94	1,190	4,260
Fumonisins	65	9	14	25	59
ZEN	65	7	11	28	89
**Rice bran meal**					
Aflatoxins	12	1	8	3	3
Trichothecenes type B	12	10	83	95	203
Fumonisins	12	4	33	385	583
ZEN	12	9	75	61	199
**Cottonseed gluten meal**					
Aflatoxins	13	3	23	4	7
Trichothecenes type B	13	1	8	22	22
Fumonisins	13	8	62	485	2,356
ZEN	13	4	31	18	23

### Occurrence of Mycotoxins in Different Maize By-Products

As presented in Table 5, maize by-products were contaminated with multiple mycotoxins. Aflatoxins were detected in 45% of samples in a sprayed corn husk, which were the highest percentage of maize by-products, reaching an average positive concentration of 45 μg/kg. Trichothecenes type B, fumonisins, and ZEN were more prevalent, detected in almost 100% of the maize by-product samples. In sprayed corn husk, averages of positive concentrations of trichothecenes type B, fumonisins, and ZEN were 8,062, 15,566, and 1,563 μg/kg, respectively. In DDGS, the average of positives of trichothecene type B was as high as 4,008 μg/kg, and the average of ZEN in corn gluten meal was also extremely high with a value of 1,643 μg/kg in this survey.

**Table 5:** Mycotoxin occurrence of different maize by-products in China in 2021.

**Mycotoxin**	**Sample number**	**Positive samples**		**Average of Positives (μg/kg)**	**Maximum (μg/kg)**
		**Number**	**%**		
**Distillers dried grains with solubles (DDGS)**					
Aflatoxins	27	3	11	25	65
Trichothecenes Type B	27	27	100	4,008	12,255
Fumonisins	27	27	100	1,607	14,217
Zearalenone (ZEN)	27	27	100	416	1,685
**Corn gluten meal**					
Aflatoxins	20	2	10	21	46
Trichothecenes type B	20	19	95	846	2,093
Fumonisins	20	20	100	5,905	30,373
ZEN	20	19	95	1,643	10,467
**Sprayed corn husk**					
Aflatoxins	11	5	45	49	157
Trichothecenes Type B	11	11	100	8,064	20,213
Fumonisins	11	11	100	15,566	41,700
ZEN	11	11	100	1,563	5,368

### Occurrence of Mycotoxins in Grasses

In silages and grasses, aflatoxins showed negative in all samples. Trichothecenes type B was frequently detected in silages and oat grass, at averages of positive concentrations of 1,536 and 2,330 μg/kg, respectively. High positive rates of ZEN were also detected in samples of silages and oat grass. Their averages were 328 and 532 μg/kg, respectively (Table 6).

**Table 6:** Mycotoxin occurrence of grasses in China in 2021.

**Mycotoxin**	**Sample number**	**Positive samples**		**Average of positives (μg/kg)**	**Maximum (μg/kg)**
		**Number**	**%**		
**Silage**					
Aflatoxins	243	0	0	0	0
Trichothecenes type B	243	209	86	1,536	13,513
Fumonisins	243	211	87	777	6,533
Zearalenone (ZEN)	243	185	76	328	8,649
**Alfalfa**					
Aflatoxins	16	0	0	0	0
Trichothecenes type B	16	1	6	71	71
Fumonisins	16	8	50	33	78
ZEN	16	4	25	76	215
**Oat grass**					
Aflatoxins	64	0	0	0	0
Trichothecenes type B	64	44	69	2,330	9,363
Fumonisins	64	40	63	386	1,986
ZEN	64	39	61	532	2,622
**Soybean hull**					
Aflatoxins	6	1	17	1	1
Trichothecenes type B	6	4	67	90	187
Fumonisins	6	5	83	41	80
ZEN	6	4	67	41	102

### Occurrence of Mycotoxins in Finished Feeds

Trends of mycotoxin occurrence were similar in swine and poultry finished feeds, while poultry feed exhibited more severe mycotoxin contamination (Table 7). In total, 21 and 33% of samples were detected with aflatoxins in swine and poultry finished feeds, respectively. Trichothecenes type B, fumonisins, and ZEN showed high prevalence in general. In poultry and swine feeds, trichothecenes type B reached average positive concentrations of 870 and 646 μg/kg, respectively, and ZEN was also detected with averages of 164 and 88 μg/kg, respectively. Fumonisins were detected at average positive concentrations of approximately 1,000 μg/kg in both feed samples. In cow concentrate supplement, trichothecenes type B and ZEN were all detected with 100% positive samples at average values of 1,091 and 127 μg/kg, respectively. In TMR, trichothecenes type B were the most prevalent at an average positive concentration of 818 μg/kg. Fumonisins and ZEN were at a relatively low mycotoxin risk level.

**Table 7:** Mycotoxin occurrence of finished feeds in China in 2021.

**Mycotoxin**	**Sample number**	**Positive samples**		**Average of Positives (μg/kg)**	**Maximum (μg/kg)**
		**Number**	**%**		
**Swine feed**					
Aflatoxins	350	74	21	4	49
Trichothecenes type B	350	347	99	646	3,620
Fumonisins	350	347	99	966	8,539
Zearalenone (ZEN)	350	336	96	88	857
**Poultry feed**					
Aflatoxins	434	139	32	14	206
Trichothecenes type B	434	430	99	870	6,176
Fumonisins	434	430	99	1,263	12,776
ZEN	434	408	94	164	1,490
**Concentrate supplement**					
Aflatoxins	34	6	18	2	3
Trichothecenes type B	34	34	100	1,091	4,251
Fumonisins	34	34	100	1,314	7,669
ZEN	34	34	100	127	400
**Total mixed ration (TMR)**					
Aflatoxins	148	15	1	4	4
Trichothecenes type B	148	138	93	818	3,750
Fumonisins	148	135	91	553	4,618
ZEN	148	135	91	87	499

### Co-occurrence of Mycotoxins

**Figure 3:**
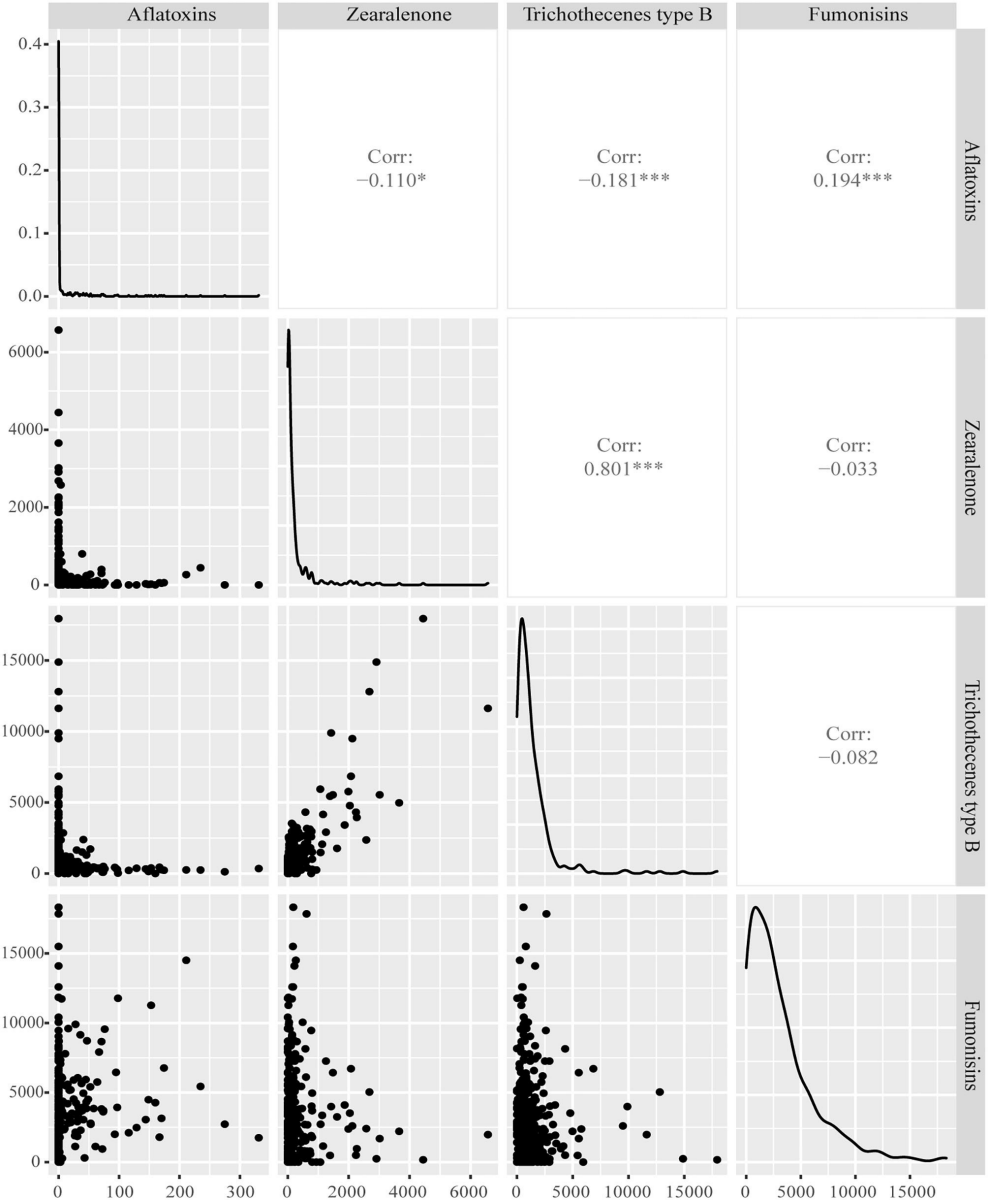
Correlation of mycotoxin concentrations of new season maize in China in 2021. Panels on the lower left side show the distribution of concentrations (in μg/kg) for each combination of two mycotoxins. Panels in the diagonal show the distribution of concentrations for each mycotoxin. Panels on the upper right side show the correlation coefficient for each combination of two mycotoxins. ***P < 0.001; **P < 0.01; *P < 0.05.

**Figure 4:**
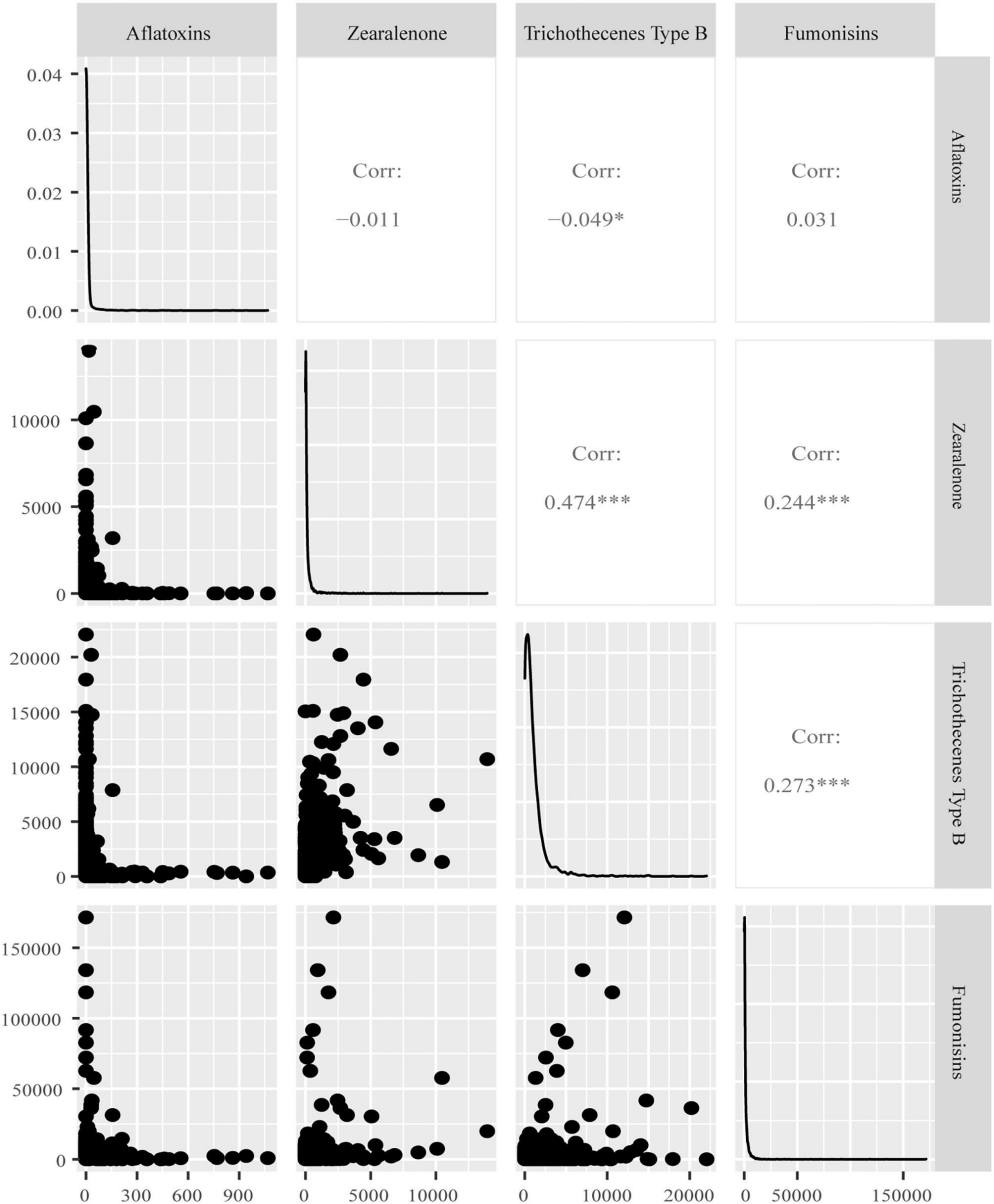
Correlation of mycotoxin concentrations of feeds and raw materials in China in 2021. Panels on the lower left side show the distribution of concentrations (in μg/kg) for each combination of two mycotoxins. Panels in the diagonal show the distribution of concentrations for each mycotoxin. Panels on the upper right side show the correlation coefficient for each combination of two mycotoxins. ***P < 0.001; **P < 0.01; *P < 0.05.

The correlations between mycotoxin concentrations for any combination of two mycotoxins were calculated to analyze the co-occurrence of major mycotoxins in samples of new season maize and feeds and raw materials of China. In Figures 3, 4, it showed that the concentrations of *Fusarium* mycotoxins trichothecenes type B and ZEN exhibit a highly significant positive correlation in the samples of both the new season maize and feeds and materials. Their coefficients were 0.801 and 0.474, respectively. Furthermore, positive correlations were observed between concentrations of fumonisins and trichothecenes type B, fumonisins and ZEN in feeds (Figure 4), and fumonisins and aflatoxins were also correlated in maize (Figure 3). In this survey, the correlations for other pairs of mycotoxins had no or low correlation coefficients.

## Discussion

In general, aflatoxins showed a relatively low positive rate in the feeds of China. Except for some seriously polluted samples of maize by-products and peanut meal, most collected samples complied with the regulated values of AFB1 of China. On the contrary, the *Fusarium* mycotoxins are more frequently detected in this study. This result has been supported by a global survey of mycotoxin contamination of feeds during a 10-year period from 2008 to 2017 (12), in which, the prevalence and concentrations of *Fusarium* mycotoxins in East Asia were even higher than in most of the other regions of the world. Considering the key roles of climate parameters on fungi infestation and their metabolite production, mycotoxins can be expected to present a similar mode of occurrence from year to year. As reported by research conducted in 2011, the prevalence of *Fusarium* mycotoxins in the contamination of feeds and materials can be traced back then in China (19). These examples illustrate that the high-risk levels of ZEN, trichothecenes type B, and fumonisins in animal feeds and materials in China are a recurring issue that deserves attention.

### Mycotoxin Occurrence in Different Feed Commodities

Each feed raw material showed a distinct pattern of mycotoxin occurrence. Maize is normally found to be frequently contaminated with DON, ZEN, and fumonisins. Wheat and bran are mainly contaminated by ZEN and DON. Peanut is easily detected with aflatoxins, and soybean usually presents a high level of ZEN infestation (12). These results can reflect well-known associations of some fungal species with certain crop plants. For example, fumonisin producer *F. verticillioides* is a pathogen of maize (20), and DON and ZEN producers *F. culmorum* and *F. graminearum* mainly infest maize, wheat, barley, and rice (21). Aflatoxins are synthesized primarily by *A. flavus* and *A. parasiticus*, and these strains are particularly prone to contaminate maize and nuts according to previous research (22).

Maize by-products exhibited enriched mycotoxins. Sprayed corn husk is a by-product of starch with com steep liquor sprayed onto the husk. According to Trenholm et al. (23), 40%−100% of DON and ZEN can be removed from maize to the husk when it is dehulled, and in this survey, trichothecenes type B, fumonisins, and ZEN concentrations were detected 6–7 times higher than in the material of maize. In DGGS, trichothecenes type B and ZEN levels were both nearly three times the levels in the original maize, and this should be mainly due to the reason that the fermentation and distillation processes of bioethanol could concentrate the previously existing mycotoxins in maize up to three times in the by-product (24). The opposite trends of aflatoxins and fumonisins in DDGS and sprayed corn husks were observed, and these results are likely to be related to the uneven distribution of sampling sites of maize by-products and the lack of samples from high-aflatoxins and high-fumonisins regions, such as East China in this survey.

*Fusarium* toxins were the main mycotoxins found in silages, and the concentrations of trichothecenes type B and ZEN were very high in individual samples of this survey. This contamination could be mainly due to the polluted raw materials obtained in the field and the post-harvest contaminants (25). Several studies have obtained similar results. DON was dominant with a positive rate of 62% in maize silages globally during a decade dataset (12), and a survey scaled on 15 European countries revealed that the maximum concentrations of trichothecenes type B and fumonisins were found in maize silages. By multiannual research focused on farms with suspected mycotoxin problems in Argentina, DON and ZEN represented a frequent threat to livestock in silages with average concentrations of 1,653 and 451 μg/kg, respectively (26). The incidence of aflatoxins is relatively low in well-preserved silages.

Poultry, swine finished feeds, and TMR showed high positive rates of all mycotoxins. This could be mainly due to the finished feeds and TMR being blended with different raw materials and then containing all mycotoxins occurring in these commodities.

### Regional Patterns of Mycotoxin Contamination in Maize

Positive rates and averages of positive concentrations of mycotoxins in maize varied among regions. Several factors can contribute to these differences. In addition to the pre- and post-harvest agricultural practices during the development and storage of crops, climate parameters varying between regions, such as temperature and rainfall, can play key roles in the growth of toxigenic fungi and mycotoxin production.

In this study, the prevalence of aflatoxins was higher in samples from Jiangsu and Anhui provinces. Aflatoxins production is dependent on various weather conditions. In the period of plant growth period, which is normally July for the maize silking period in China, *Aspergillus* spp. infestation and aflatoxins production are favored by relatively high temperature and low humidity. Later in the harvest season and storage period, aflatoxins can be increased by a combination of relatively high temperature and high precipitation (27–29). So it is widely accepted that aflatoxin contamination on crops, such as maize and peanuts, is significant in tropical and subtropical regions. On a global scale, the geographical territories of Africa, the Middle East, South Asia, and South Europe regions are all demonstrated to normally obtain high concentrations of aflatoxins (6, 30). According to this survey scaled in major maize producing provinces of China, the weather conditions feathering relatively high rainfall in the hot season may facilitate the prevalence of aflatoxins in the processes of harvest and storage of maize in Anhui and Jiangsu provinces, and this pattern should continue to be monitored closely.

Mild temperature and precipitation play key roles in contamination levels of DON (31). It has been reported that DON can be commonly detected in relatively cold and humid conditions in Northern Europe and North America (32). For several regions analyzed in this study, higher concentrations of trichothecenes type B were detected in samples from the temperate regions of Northeast, North, and East China, and these data confirm the key impact of colder weather on contamination levels of DON in maize.

In addition, ZEN was frequently observed to co-occur with DON in maize (33, 34), as DON and ZEN can be both produced by *F. graminearum* and *F. culmorum* species. ZEN showed relatively high levels in the northern regions of China, and the highest concentration was also exhibited in Shandong province in this study. This could be associated with the relatively heavy rainfall, especially in July and August of 2021 with the typhoon striking in Shandong province. Overall, special attention should be paid to the risk of DON and ZEN contamination in maize and its by-products if unusual precipitation occurs, especially in northern geographical regions.

Hot temperatures and relatively low rainfall during the maize silking period were shown to favor the fumonisins contamination (35, 36). Therefore, regions with relatively hot temperatures, such as Anhui and Jiangsu provinces, showed a high level of fumonisins contamination. In addition, the content of fumonisins in samples from northern to southern provinces of China showed a significant upward trend in this survey, which also illustrated to some extent that the increased temperature would increase the prevalence of fumonisins in maize. In a recent global mycotoxin survey (12), an extremely high fumonisins concentration was also observed in East Asia, relating to the high temperature in China's main maize production areas in the summer season of the survey-conducted year. It confirms the effect of high temperatures on fumonisins production in maize.

### Co-occurrence of Mycotoxins

Co-contamination of mycotoxins can be frequently observed, with more than three major mycotoxins in some single samples. Trichothecenes type B, represented by DON, and ZEN have been observed to frequently co-occur, especially in temperate regions. Exposure of livestock to DON and ZEN is very common and the negative effects on animal health should not be underestimated. Additive and synergistic effects of DON and ZEN have been observed on different biological parameters in different animal species. The immune function (37), the intestinal barrier function (38, 39), the liver health (40), the oxidative stress in the spleen (37), brain (41), and kidneys (42) can be affected with different levels of these toxins in mice and pigs.

In this survey, aflatoxins and fumonisins were also detected with a relatively high correlation in maize samples. This result mainly agreed with a previously published study, in which AFB1 and FB1 co-occurred in a total of 93% of maize samples within China (43). However, this phenomenon did not remain the same all the time. No co-occurrence of AFB1 and FB1 was discovered in the maize of China by Feng et al. (44), and this could be due to that aflatoxins are post-harvest and fumonisins are pre-harvest mycotoxins. Although the co-occurrence was not totally inconsistent, synergistic negative effects on performance parameters have been observed in several animal species (33). More importantly, AFB1 and fumonisins can induce liver lesions (45), suggesting a stronger toxic effect of the mycotoxin mixtures compared with each individual mycotoxin.

Due to the relatively high temperature, heavy precipitation, and frequent extreme weather, such as typhoon and flood, the risk of mycotoxin contamination of raw materials and feed commodities were increased significantly. Generally, trichothecenes type B and ZEN were dominantly detected in wheat, while trichothecenes type B, fumonisins, and ZEN were the most prevalent mycotoxins in the new season maize. The concentration levels of trichothecenes type B and ZEN were increased significantly in the main maize-producing areas of China in the year 2021 compared with the previous year. As a result, the contamination levels were also remarkably raised in the finished feeds and TMR in the year 2021. Since mycotoxin contamination in feeds and food can lead to serious health problems and economical losses, monitoring and supervision of mycotoxin contamination are very necessary. More samples representing various raw materials and production regions are needed in such a survey so as to monitor the trend more accurately. This kind of survey program also helps to raise awareness of mycotoxins. Effective mycotoxin prevention and control strategies, including the application of in-feed detoxification solutions, are needed to minimize mycotoxin contamination in feed and food.

## Data Availability Statement

The original contributions presented in the study are included in the article/supplementary material, further inquiries can be directed to the corresponding author.

## Author Contributions

WH: conceptualization, data analysis, validation, and writing—original draft and editing. AL: data analysis, investigation, and writing—original draft. JW: validation, investigation, and resources. GA: supervision, project administration, and funding acquisition. SG: writing—review and editing, supervision, and project administration. All authors contributed to the article and approved the submitted version.

## Conflict of Interest

WH, AL, JW, and GA was employed by DSM (China) Co., Ltd. SG was employed by DSM Singapore Industrial Pte Ltd.

## Publisher's Note

All claims expressed in this article are solely those of the authors and do not necessarily represent those of their affiliated organizations, or those of the publisher, the editors and the reviewers. Any product that may be evaluated in this article, or claim that may be made by its manufacturer, is not guaranteed or endorsed by the publisher.
